# Photoacoustic “nanobombs” fight against undesirable vesicular compartmentalization of anticancer drugs

**DOI:** 10.1038/srep15527

**Published:** 2015-10-20

**Authors:** Aiping Chen, Chun Xu, Min Li, Hailin Zhang, Diancheng Wang, Mao Xia, Gang Meng, Bin Kang, Hongyuan Chen, Jiwu Wei

**Affiliations:** 1 Jiangsu Key Laboratory of Molecular Medicine, Medical School and the State Key Laboratory of Pharmaceutical Biotechnology, Nanjing University, 210093, China; 2 State Key Laboratory of Analytical Chemistry for Life Science, School of Chemistry and Chemical Engineering, Nanjing University, 210093, China; 3 Nanjing University Hightech Institute at Suzhou, Suzhou, 215523, China

## Abstract

Undesirable intracellular vesicular compartmentalization of anticancer drugs in cancer cells is a common cause of chemoresistance. Strategies aimed at circumventing this problem may improve chemotherapeutic efficacy. We report a novel photophysical strategy for controlled-disruption of vesicular sequestration of the anticancer drug doxorubicin (DOX). Single-walled carbon nanotubes (SWCNTs), modified with folate, were trapped in acidic vesicles after entering lung cancer cells. Upon irradiation by near-infrared pulsed laser, these vesicles were massively broken by the resulting photoacoustic shockwave, and the vesicle-sequestered contents were released, leading to redistribution of DOX from cytoplasm to the target-containing nucleus. Redistribution resulted in 12-fold decrease of the EC_50_ of DOX in lung cancer cells, and enhanced antitumor efficacy of low-dose DOX in tumor-bearing mice. Side effects were not observed. These findings provide insights of using nanotechnology to improve cancer chemotherapy, i.e. not only for drug delivery, but also for overcoming intracellular drug-transport hurdles.

The success of an anticancer drug depends on its ability to accumulate in the local microenvironment surrounding the drug target. However, cancers exploit multiple sophisticated means to escape chemotherapy. Acquired multidrug resistance (MDR) in cancer cells is a well-known obstacle to successful therapeutic outcomes in cancer patients^1^,^2^,^3^. MDR is a multifactorial, complex process^4^,^5^. Accumulating evidence indicates that sequestration of anticancer drugs in cytoplasmic organelles (e.g., vesicles) apart from drug-targeting compartments (e.g., nucleus) contributes significantly to the MDR phenotype^6^,^7^. Mechanisms include pH partitioning within mammalian cells leading to selective compartmentalization of weak base chemotherapeutic drugs, such as anthracyclines and vinca alkaloids, into acidic vesicles^8^, and active transport of intracellular drugs into vesicles^9^,^10^. Considering that most commercially available anticancer drugs target DNA or nuclear enzymes, undesirable vesicular sequestration of anticancer drugs not only results in insufficient drug accumulation in the nucleus, but also increases drug exocytosis, both of which contribute to chemoresistance. Although some approaches aimed at reducing cytoplasmic sequestration have shown promise^11^, there is no strategy to induce tumor-targeted disruption of undesirable vesicular sequestration of anticancer drugs, while sparing normal tissues to reduce side effects.

Nanomaterials have been actively pursued in recent years in various strategies to improve chemotherapy of cancer. Multiple types of nanomaterials have been successfully used as carriers to deliver therapeutic agents to tumor tissues and cancer cells^12^,^13^,^14^, or as wrap-materials to reduce the side effects of anticancer drugs^15^,^16^. However, little has been done to confront the obstacles of undesirable subcellular drug sequestration following cellular entry.

Single walled carbon nanotubes (SWCNTs), a carbon nanomaterial with unique physical and chemical properties, have been extensively explored as carriers for delivery of chemotherapeutic drugs, proteins, genes and molecular agents^17^,^18^,^19^,^20^,^21^,^22^. When conjugated with appropriate ligands, such as folate, SWCNTs can be easily internalized into cancer cells and trapped in intracellular vesicles^23^,^24^,^25^. Because of the strong optical absorption of SWCNTs in the near-infrared (NIR) region, photothermal ablation of cancer cells harboring SWCNTs is a promising approach, and has been evaluated both *in vitro* and *in vivo*^26^. We showed previously that a large photoacoustic effect of SWCNTs under irradiation of NIR pulsed laser triggered a firecracker-like, nanoscale explosion^27^. This photoacoustic effect could be fine-tuned by modulation of laser power and pulse width, generating strong or weak explosions^28^. Irradiation using a millisecond NIR pulsed laser with high output energy produced in a high magnitude explosion and destroyed cancer cells^27^,^29^. However, we found that a relatively weak explosion could be achieved using a nanosecond laser and a much lower pulse power.

We hypothesized that SWCNTs could be used as photoacoustic “nanobombs” to modulate on-demand the undesirable vesicular compartmentalization of anticancer drugs, and hence, not only to overcome chemoresistance but also to reduce side effects of drugs on normal tissues. In the present study, we report use of finely-tuned photoacoustic “nanobombs” to abrogate chemoresistance in lung cancer through selective and spatially-controlled disruption of vesicular sequestration of the anticancer drug doxorubicin (DOX).

## Results

### Conceptual scheme and design of photoacoustic “nanobombs”

**Figure 1:**
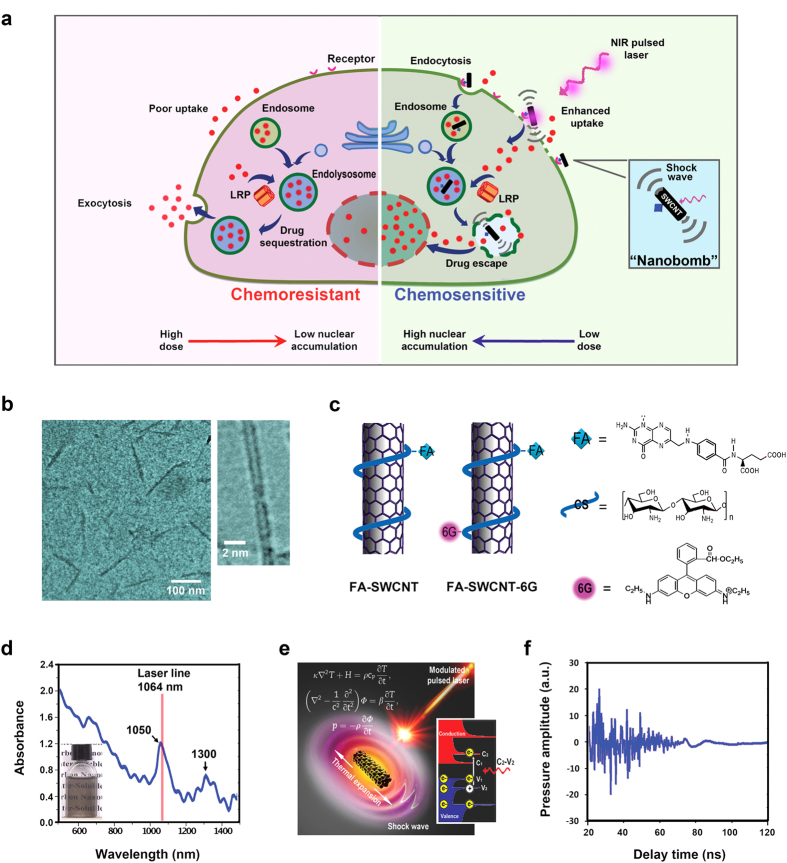
Conceptual schema and design of photoacoustic “nanobomb”. (**a**) A schema showing typical chemoresistance pathway mediated by vesicular compartmentalization of anticancer drugs (Left panel), and a proposed chemosensitizing pathway that selectively disrupts undesirable vesicular sequestration using a photoacoustic “nanobomb” (Right panel). (**b**) TEM images of the SWCNTs. (**c**) Conjugation strategy of FA-SWCNTs and FA-SWCNTs-6G. (**d**) UV-VIS-NIR absorption spectrum of the SWCNTs in water; inset shows a photograph of the solution. (**e**) Illustration of the mechanism of photoacoustic shockwave generated by SWCNTs upon laser irradiation. (**f**) Generation of a photoacoustic signal after irradiation with a single laser pulse at a width of 5 ns and a power of 0.25 mJ per pulse.

The concept of this novel strategy is depicted in Fig. 1a. The left panel shows typical chemoresistance pathways by which nucleus-targeting drugs are sequestered by vesicular compartmentalization; the right panel shows a designed pathway to induce chemosensitivity by breakdown of undesirable vesicular sequestration using photoacoustic “nanobombs”. The “nanobombs” are initiated from purified single-walled carbon nanotubes (SWCNTs) produced using a chemical vapor deposition method, having an average length of 100–200 nm and a diameter of 2 nm (Fig. 1b). To improve their biological capabilities and to further modify their performance characteristics, the SWCNTs were conjugated with chitosan oligomer (CS) using a non-covalent approach^23^,^27^. Folic acid (FA) was then covalently coupled to the chitosan coated SWCNTs. The folate enables the nanobomb to specifically interact with folate receptors commonly overexpressed on most cancer cells^23^,^24^,^25^, thereby, aiding the cellular internalization of the nanotubes (Fig. 1c). To dynamically track the biodistribution of SWCNTs in cells, the FA-SWCNT were further labeled with a fluorescent rhodamine-6G (FA-SWCNT-6G) (Fig. 1c). The functionalized carbon nanotubes exhibited monodispersity in aqueous solution, and had strong absorbance around 1050 and 1300 nm (Fig. 1d). These two absorption bands originated from the electronic transition between the first or second Van Hove singularities of nanotubes (Fig. 1e). The Van Hove-like singularities enhance the effective density of states near the Fermi energy and increase the electron–phonon interaction, thereby increasing the temperature of the nanotube. Upon irradiation by pulsed laser at a wavelength of 1064 nm using a pulse width of 5 nanoseconds and pulse energy of 0.25 mJ, an acoustic shockwave was generated from the carbon nanotube (Fig. 1f).

### Photoacoustic “nanobombs” selectively disrupt acidic vesicles

**Figure 2:**
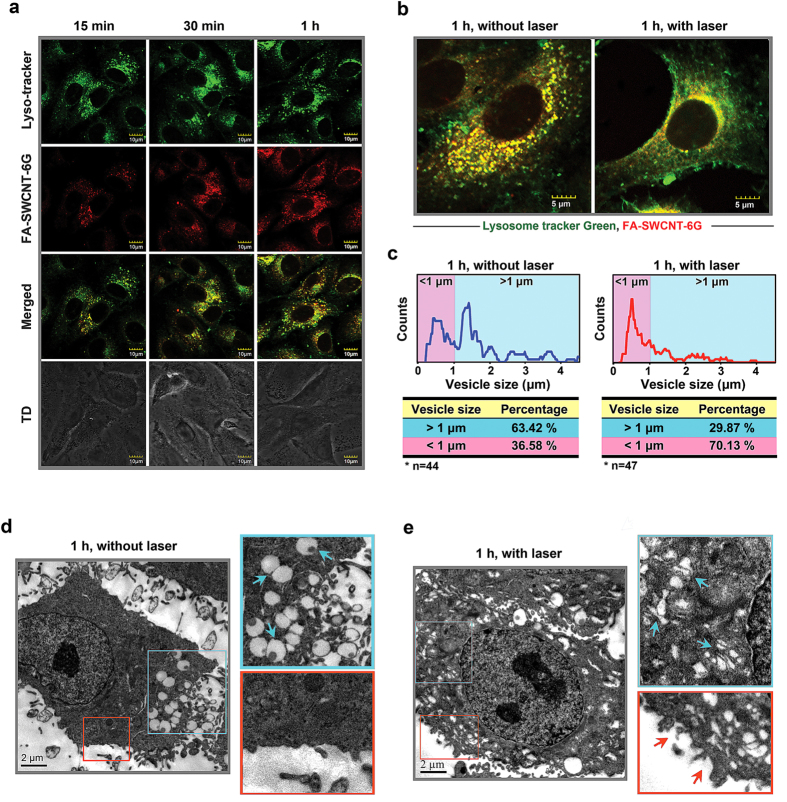
Photoacoustic “nanobombs” selectively disrupt acidic vesicles. (**a**) A549 cells were pretreated with LysoTacker Green (green dots) for 1 h followed by incubation with FA-SWCNT-6G (red dots). The dynamic intracellular distribution was monitored by confocal fluorescence microscopy at 15, 30, and 60 min after FA-SWCNT-6G incubation. Yellow puncta depict colocalization of Lysotracker (green) and FA-SWCNT-6G (red) dots. The bottom panel shows the phase contrast of A549 cells. Scale bars = 10 μm. (**b,c**) A549 cells were pretreated with LysoTracker Green for 1 h followed by incubation with FA-SWCNT-6G for another hour. Cells were then irradiated or not for 15 min, and (**b**) subjected immediately to confocal microscopy. Scale bars = 5 μm. (**c**) Intracellular vesicles were quantitatively ranked by size (<1μm or >1 μm) in cells without (left panel, n = 44) or with irradiation (right panel, n = 47). The percentage of each distribution is shown. (**d,e**) A549 cells were incubated with SWCNTs for 1 h and then treated with laser for 15 min. TEM showing vesicle morphology of cells before (**d**) and after (**e**) laser irradiation. Scale bar = 2 μm. Enlarged images show intracellular vacuoles (cyan arrows), and perforation of plasma membranes (orange arrows).

We have previously shown that folate-conjugated carbon nanotubes could be internalized by cancer cells through receptor-mediated endocytosis^23^,^24^,^25^,^27^. Here, we further investigated the dynamic biodistribution of nanotubes taken up by cancer cells. We found that fluorescent dye-labeled SWCNTs (FA-SWCNT-6G) were largely sequestered into vesicles within 15 min of incubation with A549 cells. Furthermore, the FA-SWCNT-6G-containing vesicles were increasingly co-localized with lysosomes after 30 to 60 min, indicating that FA-SWCNT-6G were internalized and then trafficked into lysosomes (Fig. 2a).

We then wanted to know if the fine-tuned photoacoustic effect of SWCNTs could selectively disrupt the vesicular trafficking upon laser irradiation. We found that SWCNTs that were compartmentalized within acidic vesicles after entering cells were released into the cytosol after laser irradiation (Fig. 2b), suggesting that these acidic vesicles were broken by the photoacoustic effects of SWCNTs. Further evidence that this was the case was provided by quantitative analysis of intracellular vesicles before and after laser irradiation, which showed that the distribution patterns of vesicle sizes were dramatically changed (Fig. 2c). We found that about 63.42% of intracellular vesicles were larger than 1 μm before laser irradiation, and this percentage was reduced to 29.87% after laser irradiation. Conversely, smaller vesicles with a size less than 1 μm were increased from 36.58% to 70.13% after irradiation. We further assessed this phenomenon by TEM. We found that before laser irradiation most vesicles exhibited an intact spherical morphology and were about 1 μm in diameter (Fig. 2d); however, following irradiation, they were morphologically transformed into cracked and fragmented vesicles of smaller size (Fig. 2e). In addition to breakdown of intracellular vesicles, discontinuous punctiform structures appeared on the cell membrane of laser-irradiated cells, suggesting that the membrane was possibly perforated by the shockwave from the “nanobomb” (Fig. 2e). Taken together, these results indicate that laser-induced detonation of targeted photoacoustic “nanobombs” selectively disrupt acidic vesicles in which they were internalized.

### Photoacoustic “nanobombs” facilitate nuclear accumulation of anticancer drugs

**Figure 3:**
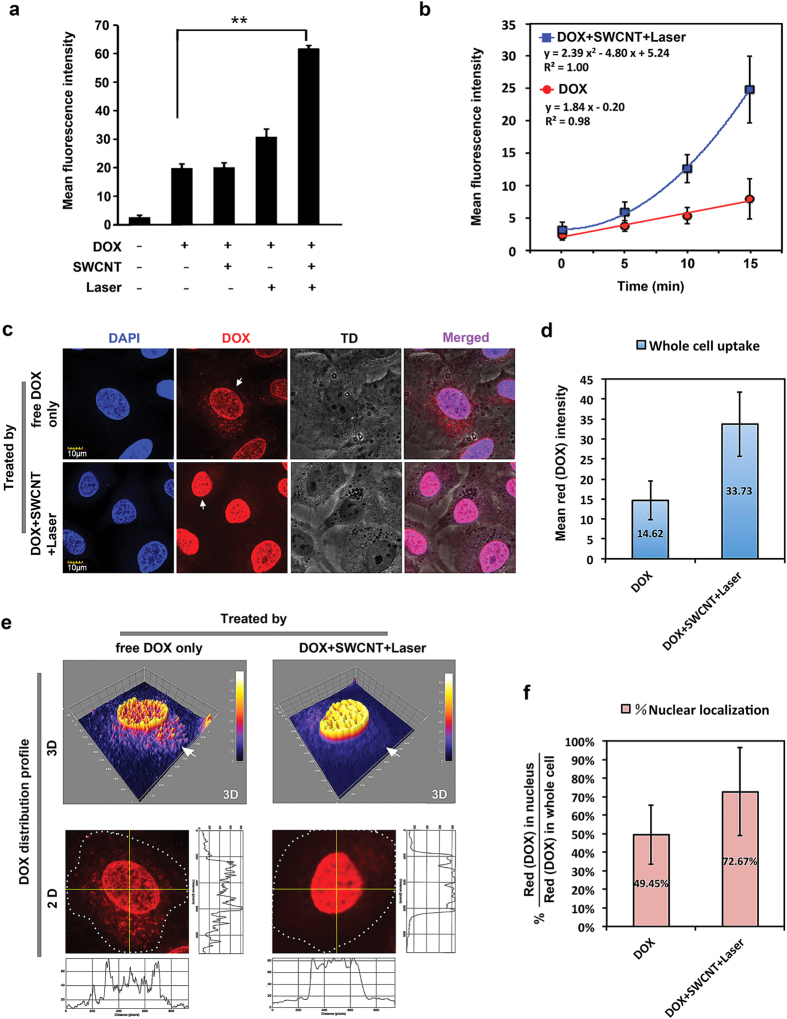
Photoacoustic “nanobombs” facilitate accumulation of DOX into the nucleus. (**a**) A549 cells were incubated with or without SWCNTs for 1 h followed by administration of DOX and/or laser irradiation for 15 min. The uptake of DOX in cells was determined by flow cytometry. Untreated cells were used as control. Means+SD. of three independent experiments are shown. **P < 0.01. (**b**) A549 cells were incubated with or without SWCNTs for 1 h followed by DOX treatment. Cells were irradiated (or not) at serial time points. Time-dependent dynamics of DOX uptake were then measured by flow cytometry. Means ±SD of three independent experiments are shown. (**c**) A549 cells were incubated with SWCNTs for 1 h and further incubated with DOX followed by laser irradiation for 15 min. Cells incubated with DOX alone were used as controls. Intracellular uptake of DOX was observed by confocal fluorescence microscopy. The nucleus was stained with DAPI (blue). Scale bars = 10 μm. (**d**) Quantitative analysis of the mean fluorescence (red) intensity in cells treated as described in (**c**). Means ± SD are shown (n = 30). (**e,f**) Intracellular biodistribution of DOX monitored by confocal microscopy in cells treated with SWCNT/DOX/Laser or free DOX only. (**e**) Image analysis of subcellular distribution of DOX. (**f**) Quantification of the ratio of nuclear/whole cell DOX. Means ± SD are shown (n = 30).

The weak base anticancer drug DOX is an inhibitor of topoisomerase II, which is confined within the nucleus^30^. Cytoplasmic sequestration of DOX within acidic vesicular compartments limits accumulation in the nucleus resulting in chemoresistance^9^,^31^. In view of our data showing that photoacoustic “nanobombs” selectively disrupted acidic vesicles and possibly perforated the cell membrane, we wanted to determine whether these “nanobombs” could aid escape of DOX from vesicular sequestration and facilitate accumulation in the nucleus. We found that cellular uptake of free DOX in A549 cells treated with SWCNT/Laser was about 3 fold higher than in cells either treated with DOX alone or with SWCNT without laser irradiation (Fig. 3a). Moreover, uptake of free DOX exhibited different dynamics in cells treated with SWCNT/Laser; i.e., the uptake of free DOX showed linear dynamics, indicating that uptake speed was constant at a given concentration. However, uptake of free DOX by cells treated with SWCNT/Laser showed second order non-linear dynamics; i.e., uptake speed was a non-linear function of laser irradiation time (Fig. 3b). This result confirmed that “nanobombs” might perforate the cytoplasmic membrane and, thus, accelerate drug uptake.

We next investigated the influences of photoacoustic “nanobombs” on intracellular drug distribution. Whole-cell uptake and the nuclear localization of DOX were quantified based on confocal imaging. Whole-cell DOX uptake by cells treated with SWCNT/laser was about 2.3 times higher than cells treated with DOX alone (Fig. 3c,d), which is comparable to the extent of uptake determined by flow cytometry in Fig. 3a. Moreover, while 50.55% of total intracellular DOX was sequestered within cytoplasmic vesicle-like spots in cells treated with DOX alone, 72.67% of total intracellular DOX accumulated within the nucleus of cells treated with SWCNT/Laser (Fig. 3e,f). These results suggest that photoacoustic “nanobombs” effectively promote drug uptake and liberate drug from cytoplasmic vesicles to the nucleus.

### Photoacoustic “nanobombs” amplify therapeutic efficacy of free DOX both *in vitro* and *in vivo*

**Figure 4:**
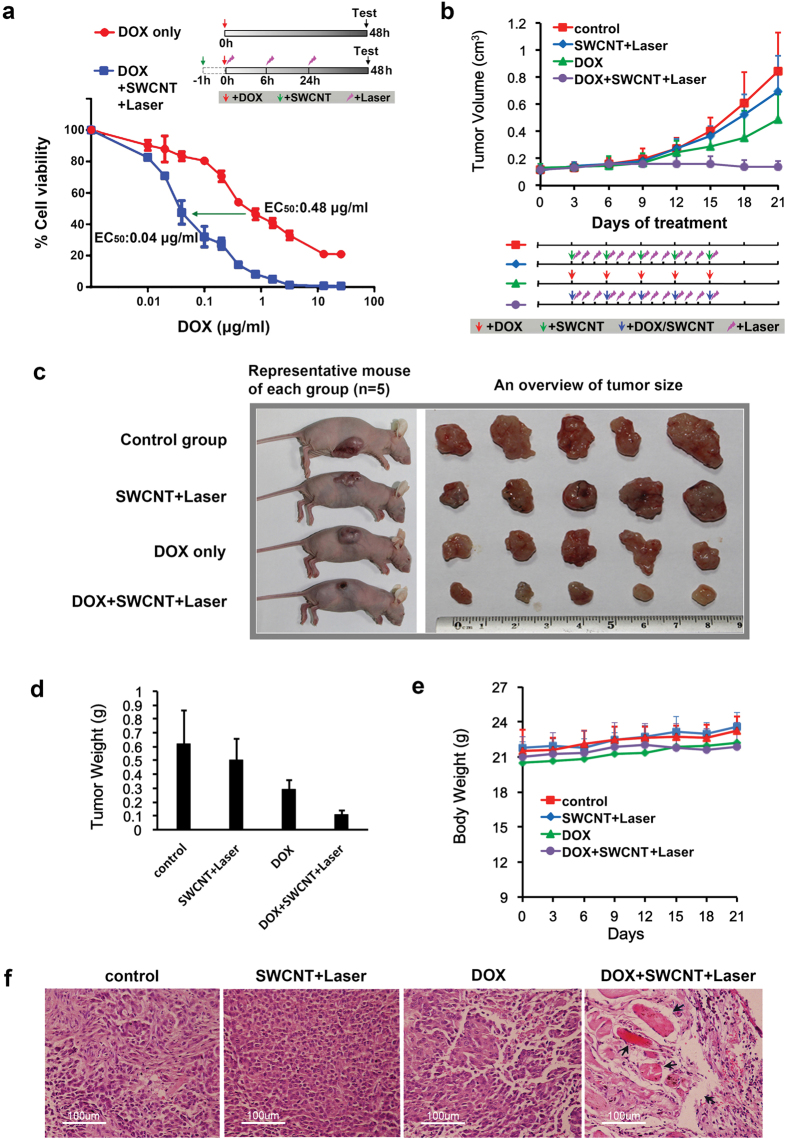
Photoacoustic “nanobombs” amplify therapeutic efficacy of DOX *in vitro* and *in vivo.* (**a**) *In vitro* cytotoxicity of DOX with and without SWCNTs/laser. Means ± SD are shown (n = 6). The laser irradiation was 15 min at 100 mW cm^−2^, and the SWCNT concentration was 10 μg ml^−1^. (**b**) The growth curves of A549 tumors on mice receiving different therapeutic formulations. Means + SD are shown (n = 5 per group). The laser irradiation was 1 min at 600 mW cm^−2^, and the SWCNT concentration was 1.5 mg kg^−1^. (**c**) Representative images of A549 tumor-bearing mice (left panel) and the separated tumor tissues after treatment (right panel). (**d**) The weight of tumor tissues after different treatments. Means + SD are shown (n = 5 per group). (**e**) Body weight variation of the mice during the treatment. Means + SD are shown (n = 5 per group). (**f**) Tumor sections were processed with hematoxylin (for nuclear staining, dark purple dots) and eosin (for cytoplasm counterstaining, deep pink). Black arrows head depict necrotic areas containing less or no cells. Scale bars = 100 μm.

We wanted to determine whether reduced vesicular sequestration and enhanced nuclear accumulation of free DOX mediated by SWCNT/Laser could amplify therapeutic efficacy. We found that the half maximal effective concentration (EC_50_) of DOX was reduced about 12 fold in A549 cells treated with SWCNT/Laser (0.04 μg ml^−1^) compared to cells treated with DOX alone (0.48 μg ml^−1^) (Fig. 4a). Of note, there was no significant cytotoxicity of SWCNT/Laser treatment, as shown by the data points of DOX = 0 μg ml^−1^ in Fig. 4a. We further investigated the feasibility of recruiting photoacoustic “nanobombs” to enhance therapeutic outcomes of low-dose DOX (to minimize toxicity) in tumor-bearing mice. While DOX single therapy exerted a mild antitumor effect, tumor growth was remarkably inhibited in mice treated with DOX/SWCNT/Laser (Fig. 4b–d). No obvious side effects were observed during the treatment as evidenced by comparable body weight among the four treatment groups (Fig. 4e). Consistently, as determined by histological examination in tumor tissues dissected from mice, we found that cell density was reduced; bleeding and necrotic areas were increased in tumors treated with DOX/SWCNT/Laser compared to other groups (Fig. 4f). These results indicate that photoacoustic “nanobombs” enhanced the therapeutic efficacy of free DOX both *in vitro* and *in vivo*.

## Discussion

Undesirable intracellular compartmentalization of anticancer drugs leads to chemoresistance. In this study, we show that photoacoustic “nanobombs” can be used to overcome undesirable vesicular sequestration of anticancer drugs resulting in significantly improved therapeutic efficacy. Our study provides a proof-of-principle strategy that may be suitable for any therapeutic limited by vesicular sequestration.

We found that the cytoplasmic distribution of SWCNTs was sporadic rather than diffuse after entering cells, suggesting that they were trapped in intracellular vesicles. This phenomenon was observed in our previous studies^23^,^24^,^25^,^27^, although it was not determined what type of vesicles were involved. Here we determined that most SWCNTs were trapped in acidic vesicles within an hour. Once trapped within vesicles, SWCNTs can be used as “nanobombs”, which effectively rupture the vesicular compartment when treated with controlled NIR laser irradiation employing nanosecond pulses. We have previously shown that SWCNT treated with millisecond pulsed NIR laser irradiation resulted in cell catastrophe. However, the nanosecond pulsed, low power, NIR laser irradiation used here had only a mild effect on cell viability. These “nanobombs” disrupted the acidic vesicles in which DOX was predominantly sequestered resulting in massive escape of DOX from intracellular vesicles and accumulation of DOX in the nucleus. This resulted in increased chemosensitivity and enhanced therapeutic effects of DOX even at a relatively low dose. It is likely that the disrupted vesicle trafficking produced by “nanobombs” would also block vesicular exocytosis of DOX. This effect might further facilitate redistribution of DOX into the nucleus, also contributing to enhanced antitumor efficacy.

In addition to increased redistribution of DOX to the nucleus, we also found that intracellular uptake of DOX was increased and accelerated by the photoacoustic “nanobombs”. The enhanced drug influx might be due to cytoplasmic membrane perforation induced by *in situ* explosion of SWCNTs located on the cell membrane, which would be consistent with a previous report using carbon nanoparticles and femtosecond laser pulses^32^. Based on these observations, our strategy for cancer-targeted modulation appears to be promising. Because folate receptor is often highly expressed on cancer cells, folate modified SWCNTs may preferentially bind to cancer cells over normal cells. Thus, the influx of cytotoxic drug may be primarily confined to cancer cells, which would reduce cytotoxicity to normal cells. In addition, the disturbed sequestration of drug into acidic vesicles may further improve its nuclear accumulation.

The photoacoustic strategy controlled by laser irradiation described here has several distinct advantages compared to using chemical (e.g., small molecule drugs) or biological (e.g., gene silencing) formulations. For example, spatially controlled laser irradiation of the tumor mass *in situ* would disturb vesicular sequestration of the cytotoxic drug only in tumor cells and cells within the tumor microenvironment. Vesicle sequestration would not be interrupted in normal cells outside the tumor mass, thus, decreasing systemic side effects. Moreover, as the photoacoustic effect disrupts acidic vesicles immediately upon laser irradiation, this intervention could be administered on-demand to achieve maximum absorption of anticancer drugs within the tumor mass.

Taken together, the results of this study provide insights relevant to future improvements in cancer chemotherapeutic strategies using nanotechnology. Among these are strategies to successfully deliver drugs to tumor sites or cancer cells, and to efficiently overcome intracellular hurdles that otherwise block effective transport of the drug to its target-containing compartments.

## Methods

### SWCNTs conjugation

CVD SWCNTs (10 mg) (Xianfeng Co.) were sonicated in a solution containing 30 ml H_2_SO_4_ (98%) and 10 ml HNO_3_ (65%) for 24 h. The material was washed and dissolved in chitosan (CS, M = 4000–6000, Sigma-Aldrich) solution (1:2 w/w) with sonication. The mixture was centrifuged at 15,000 g for 1 h to remove aggregates, and the supernatant was dialyzed through a 10 kDa molecular-weight cut-off (MWCO) membrane (Millipore Amicon). The concentration of CS/SWCNTs was determined by UV-vis-NIR spectroscopy. For conjugation of folate, 100 μl of folate (1 mM in PBS) was added to 10 mL CS/SWCNTs solution (1 mg ml^−1^), and then 50 μl of 1 mM 1-ethyl-3-(3-dimethylaminopropy) carbodiimide hydrochloride (EDC, Invitrogen) and 1 mM N -hydroxysulfosuccinimide (sulfo-NHS, Invitrogen) were added. For fluorescent labeling, 100 μl of rhodamine-6G (100 μM in PBS) were added to the FA-SWCNTs together with 10 μl EDC and 10 μl sulfo-NHS and incubated for 24 h. The final solution was dialyzed three times to remove unconjugated molecules.

### Laser irradiation

A 1064 nm nanosecond laser was used. The laser pulse width was about 5 ns, with a modulation frequency of 2 KHz. For *in vitro* experiments, the laser beam was adjusted by a concave lens to cover the cell cuvette (about 4.5 cm^2^) with a power density of 100 mW cm^−2^. For *in vivo* experiments, subcutaneous tumors were directly irradiated for 1 min with a power density of approximately 600 mW cm^−2^.

### Confocal microscopy

For assessing dynamic intracellular distribution, cells were stained with 1 μM LysoTracker Green (Invitrogen) 1 h at 37 °C in culture medium. The medium was then replaced with fresh medium containing FA-SWCNTs-6G for 15 min, 30 min, or 1 h, as indicated. For vesicle disruption experiments, LysoTracker staining was performed as above, and cells were incubated with FA-SWCNTs-6G for 1 h. Cells were then irradiated or not. Cells were observed using a confocal microscope (Olympus), and images were obtained using a digital camera with FV10-ASW software and analyzed using the Image J software. The average vesicle size was quantified in more than 40 cells for each condition.

For drug uptake experiments, cells were incubated with or without SWCNTs for 1 h, and then DOX was added to the medium followed by immediate laser irradiation for another 15 min. Cells were then fixed in 4% paraformaldehyde for 30 min, and cell nuclei were counterstained with 4′, 6-diamidino-2-phenylindole (DAPI, Invitrogen) prior to confocal microscopy. DOX fluorescence intensity in nuclei was quantified using ImageJ software in more than 30 different cells.

### *In vivo* antitumor efficacy

Male Balb/c nude mice (6–8 week old) were injected subcutaneously with 5 × 10^6^ A549 cells in the right flank on day 0 and randomized to 4 groups (5 mice per group). When tumor size reached a volume of about 150 mm^3^, mice were given intratumoral injections of PBS, DOX (0.5 mg kg^−1^), SWCNTs (1.5 mg kg^−1^), or SWCNTs+DOX every three days. The mice receiving SWCNTs or SWCNTs+DOX also were irradiated (600 mW cm^−2^) for 1 min immediately after the injection and again each day for two more days. Five consecutive treatment cycles were performed. The tumor size was measured by caliper every three days, and the tumor volume was calculated as length × width^2^/2. During the therapeutic period, physical examinations including body weight and behavior were performed regularly. At Day 21, the mice were euthanized, and the tumors were collected and weighed. All animal work was approved by the Animal Care Committee of Nanjing University in accordance with Institutional Animal Care and Use Committee guidelines.

### Photoacoustic measurement, Cell culture, MTT assay, Flow cytometric analysis, TEM and Histological staining

Detailed experimental procedures are provided in Supplementary Information.

## Additional Information

**How to cite this article**: Chen, A. *et al.* Photoacoustic “nanobombs” fight against undesirable vesicular compartmentalization of anticancer drugs. *Sci. Rep.* **5**, 15527; doi: 10.1038/srep15527 (2015).

## Supplementary Material

Supplementary Information
