# Supplementary material for: Photoacoustic “nanobombs” fight against undesirable vesicular compartmentalization of anticancer drugs
Source: Sci Rep. 2015 Oct 20;5:15527. doi: 10.1038/srep15527 (PMC4612315; doi:10.1038/srep15527)
Supplement: Supplementary Information [file srep15527-s1.doc]

**Supplementary Information**

**Photoacoustic “nanobombs” fight against undesirable vesicular compartmentalization of anticancer drugs**

Aiping Chen^1,3^, Chun Xu^1^, Min Li^1,3^, Hailin Zhang^1^, Diancheng Wang^1^, Mao Xia^1^, Gang Meng^1,3^, Bin Kang^2*^, Hongyuan Chen^2^, Jiwu Wei^1,3*^

^1^Jiangsu Key Laboratory of Molecular Medicine, Medical School and the State Key Laboratory of Pharmaceutical Biotechnology, Nanjing University, 210093, China

^2^State Key Laboratory of Analytical Chemistry for Life Science, School of Chemistry and Chemical Engineering, Nanjing University, 210093, China

^3^ Nanjing University Hightech Institute at Suzhou, Suzhou, 215523, China

^*^ Correspondence and requests for materials should be addressed to J.W. (email: wjw@nju.edu.cn) or to B. K. (email: binkang@nju.edu.cn)

**Experimental details:**

**Photoacoustic measurement.** The generation of photoacoustic signals on SWCNTs under laser irradiation was measured on a lab-configured setup. Briefly, a nanosecond pulsed laser (from Changchun New Industries Optoelectronics Technology Co.) was used to excite the photoacoustic effect. The laser wavelength is at 1064 nm, and the pulse width was about 5 ns with a modulation frequency of 2 KHz. The SWCNT solution with concentration about 1 mg ml^-1^ was placed in a small quartz cell, the acoustic signals were recorded by a small piezoelectric microphone, and then the pressure signals were amplified through a lock-in amplifier and further analyzed via a FFT Spectrum Analyzers (SRS Co.).

**Cell culture.** Human lung adenocarcinoma cell line A549 was obtained from American Type Culture Collection (CCL-185). Cells were maintained in Dulbecco’s modified Eagle medium (DMEM) supplemented with 0.1 mM nonessential amino acids, 5% fetal bovine serum, and 100 units ml^-1^ penicillin-streptomycin (all from Invitrogen) at 37 °C in humidified air containing5% CO_2_.

**In vitro cytotoxicity assay.** A549 cells (8×10^3^ cells per well) were seeded in 96-well plates overnight. Then cells were incubated with or without SWCNTs (10 μg ml^-1^) for 1 h followed by treatment of DOX at a serial of concentration with or without laser irradiation at 100 mW cm^-2^ for 15 min. Cells were irradiated repeatedly at the point of 6 h and 24 h. 48 h later, 20 μl per well of the MTT solution (Invitrogen) were added into the medium. After 4 h of incubation, the medium was removed, and cells were mixed with 100 μl isopropyl alcohol. The absorbance was measured at 570 nm.

**Flow cytometric analysis.** Cells were grown on 12-well plates and incubated with SWCNTs for 1h. Then DOX was added followed by irradiation with or without laser for 15 min (100 mW cm^-2^). Untreated cells or DOX treated alone were used as control. The mean fluorescence intensity was determined using a FACS Calibur (BD). DOX mass was quantified using mean fluorescence intensity averaged from 3 independent experiments.

**TEM of cells.** A549 cells were seeded in 6-well plates (3 x 10^5^ cells per well) overnight, then cells were incubated with SWCNTs for 1 h followed by laser irradiation for 15 min or left unirradiated. Cells were then harvested and frozen under high pressure, dehydrated, and chemically fixed. Ultrathin section were cut and stained with uranylacetate and lead citrate. Cells were imaged using a JEM-1011 Electron Microscopy.

**Histological staining.** Tumors were dissected, fixed in 4% paraformaldehyde,embedded in paraffin, and serially cut into 5 μm thick sections. Deparaffinized and rehydrated sections were stained with hematoxylin and eosin and observed with a microscopy.
